# Copper isotope fractionation and copper-to-zinc ratios reveal altered metal homeostasis in prostate cancer

**DOI:** 10.1038/s41598-026-46224-3

**Published:** 2026-04-20

**Authors:** Robert J. Raad, Shun-Chung Yang, Justin L. Wang, Jeremy J. Rosenbaum, Jorge Nieva, Anand Kolatkar, Stephanie N. Shishido, William M. Berelson, A. Joshua West, Peter Kuhn, Seth G. John

**Affiliations:** 1 Keck School of Medicine, University of Southern California, Los Angeles, CA USA; 2 Department of Earth Sciences, University of Southern California, Los Angeles, CA USA; 3 Department of Medicine, Keck School of Medicine, University of Southern California, Los Angeles, CA USA; 4 Michelson Center for Convergent Bioscience, Convergent Science Institute in Cancer, University of Southern California, Los Angeles, CA USA; 5 Catherine & Joseph Aresty Department of Urology, Keck School of Medicine, Institute of Urology, University of Southern California, Los Angeles, CA USA; 6 Norris Comprehensive Cancer Center, Keck School of Medicine, University of Southern California, Los Angeles, CA USA; 7 Department of Biomedical Engineering, Viterbi School of Engineering, University of Southern California, Los Angeles, CA USA; 8 Department of Aerospace and Mechanical Engineering, Viterbi School of Engineering, University of Southern California, Los Angeles, CA USA; 9 Department of Biological Sciences, Arts, and Sciences, Dornsife College of Letters, University of Southern California, Los Angeles, CA USA

**Keywords:** Stable isotope analysis, Trace metal metabolism, Multi-collector inductively coupled plasma mass spectrometry, Castration-resistant prostate cancer, Metallomics, Biochemistry, Biomarkers, Cancer, Environmental sciences

## Abstract

Trace metal homeostasis is increasingly recognized as a reflection of cellular metabolism and redox balance. This study applies high-precision copper isotope and elemental ratio analysis to human serum to investigate how prostate cancer alters systemic metal regulation. Using multi-collector inductively coupled plasma mass spectrometry (MC-ICP-MS) adapted from geochemical applications, we quantified copper isotope fractionation and Cu/Zn ratios across disease stages. Patients with localized or newly metastatic prostate cancer exhibited isotopically heavier serum copper compared to healthy controls, while advanced, castration-resistant disease showed higher copper concentrations, lower zinc levels, and elevated Cu/Zn ratios. Together, these findings suggest that prostate cancer-associated alterations in copper and zinc homeostasis may reflect modulation by sex steroid-dependent physiological states. They also demonstrate the sensitivity of MC-ICP-MS in detecting subtle isotopic shifts in biological samples and establish its value as a tool for characterizing metabolic and redox alterations in prostate cancer.

## Introduction

The analysis of divalent metal cations has long been a cornerstone of oceanographic and geochemical research, where trace metal concentrations and isotope ratios provide powerful insights into redox conditions, biological productivity, and biogeochemical cycling^1^. Techniques originally developed to quantify isotope fractionation in seawater, namely multi-collector inductively coupled plasma mass spectrometry (MC-ICP-MS), have recently been adapted to study human physiology, where similar elemental fractionation processes reflect metabolic activity and disease states. Our group has previously described a detailed method for high-precision isotope measurement in human serum^2^. The translation of these tools from environmental to biological systems enables the exploration of how isotopic variation of essential metals encodes physiological information at the molecular level.

Among essential transition metals, copper (Cu) is particularly notable for its role as an essential micronutrient and redox catalyst. With 50–150 mg total Cu present in nearly all tissues of the human body, Cu participates in electron transport, oxidative stress regulation, and iron metabolism through its incorporation into cuproenzymes such as cytochrome *c* oxidase and superoxide dismutase 1^3^. Its homeostasis is tightly controlled by a network of transporters and metallochaperones, including COX17 and ATOX1, as well as serum carrier proteins such as ceruloplasmin and albumin^4^. Uptake takes place in the intestinal cells, followed by transport to the liver for storage. Perturbation in these pathways alters both total Cu concentration and isotopic composition, as it is well known that metabolic processes preferentially utilize copper-65 (^65^Cu) or copper-63 (^63^Cu) depending on the binding environment, following thermodynamic principles^5^. Copper metabolism is further interlinked with that of zinc (Zn), as both metals share transport and storage systems, including metallothionein. Zn exerts anti-inflammatory and antioxidant effects, while Cu excess or imbalance can enhance oxidative stress; consequently, the Cu/Zn ratio reflects systemic redox and inflammatory status^6^.

Given this extensive role in metabolism, the use of copper isotope fractionation and elemental metal ratios as disease biomarkers has been previously studied. A landmark pilot study in 2015 took serial measurements of the copper isotope fractionation (δ^65^Cu/δ^63^Cu or δ^65^Cu) in the sera of 20 breast cancer and 8 colorectal cancer patients and compared them to 50 healthy donors. They found that these patients frequently exhibited lower δ^65^Cu than controls, with a significant difference noted at a δ^65^Cu threshold value of − 0.35‰, reflecting a poorer survival subgroup^7^. Subsequent work has extended these observations across several cancer types. In thyroid carcinoma, plasma δ^65^Cu is similarly decreased while tumor tissue is enriched in ^65^Cu, suggesting tissue-specific fractionation driven by altered redox and metabolic fluxes^8^. Comparable trends have been observed in ovarian cancer, where patients display lower serum δ^65^Cu and higher tumor tissue signatures than in healthy controls, as well as in hematological malignancies, where lower plasma δ^65^Cu correlates with poorer prognosis and decreased overall survival^9,10^. Overall, observations in cancer and other diseases like cirrhosis present a decrease in δ^65^Cu in the diseased state, with median values ranging from − 0.52 to 1.14‰^5,8,9,11–13^. These collective findings indicate that cancer-associated metabolic reprogramming and redox imbalance can leave a measurable isotopic and compositional fingerprint in blood and tissue, analogous to environmental isotopic signatures observed in perturbed ecosystems.

Several mechanisms have been proposed to explain the altered copper isotope signatures observed in malignancy, but without consensus. These proposed mechanisms fall broadly into two categories: tumor-intrinsic effects, which influence copper utilization within cancer cells, and systemic processes, including copper handling by major organs such as the liver and systemic inflammatory responses. Telouk et al.^7^ attribute isotopically lighter serum signatures observed in malignancy to the Warburg effect, wherein cancer cells favor anaerobic glycolysis and lactate production as a source of energy even under normoxic conditions. The resulting increase in Cu(I)-lactate complexes thermodynamically favors binding to ^65^Cu, sequestering it into tumor tissue and driving ^63^Cu into circulation, creating the observed signatures. Bondanese et al.^12^ have proposed that the hypoxic environment in tumor cells induces copper uptake by upregulating CTR1, the major copper importer. However, a model produced by Miaou and Tissot^14^ found that this mechanism is unlikely to produce fractionation to a degree measurable in the serum. They propose that metabolic changes in liver function can generate a ∼1‰ isotope fractionation during Cu uptake, explaining the ∼0.2‰ change in cancer patients. Beyond tumor-driven processes, systemic and organ-level factors have also been implicated, including sequestration into the liver and insufficient biliary excretion^15^. This challenges the notion that δ^65^Cu shifts originate solely from tumor activity. There remains the possibility that localized metabolic reprogramming, systemic distribution changes, or both contribute to the isotopic fingerprint of malignancy.

Multiple studies have shown that the Cu/Zn ratio is increased in patients with breast, colorectal, lung, and prostate cancers compared with healthy controls, and may correlate with disease stage or aggressiveness. In prostate cancer, this was a decrease in serum zinc and an increase in copper, leading to heightened Cu/Zn ratios^16,17^. This association is thought to arise from increased Cu mobilization during angiogenesis and tumor growth, coupled with depletion of Zn-dependent antioxidant defenses^18,19^. While not disease-specific, the Cu/Zn ratio provides a complementary index of redox imbalance that may enhance the interpretation of isotopic and concentration-based metal biomarkers.

The present study applies high-precision Cu isotope analysis, originally developed in the context of ocean geochemistry, to human blood samples from patients with varying stages of prostate cancer for the first time. Cu/Zn ratio is also measured across stages. By quantifying copper fractionation alongside elemental ratios, this work aims to elucidate whether cancer-associated metabolic alterations produce measurable differences in Cu isotope composition and Cu/Zn ratios in human blood.

## Methods

### Clinical samples

This study received approval from the University of Southern California Institutional Review Board for all protocols under which samples were collected (NA00054180, NA00087094, UP-17-00883, UP-16-00691). All methods were performed in accordance with the relevant guidelines and regulations.

Deidentified patient samples were obtained with informed consent prior to collection. Samples from individuals with no known pathology were procured from Scripps Normal Blood Donor Services (San Diego, California, USA) and analyzed for a comparative analysis. Peripheral blood samples were collected using standard venipuncture techniques into Streck cell-free DNA blood collection tubes and gently inverted several times to ensure anticoagulation. All samples were shipped at room temperature to the University of Southern California for processing within 48 h of collection^20–22^.

Whole blood was subjected to a two-step centrifugation protocol to separate the acellular (plasma) and cellular fractions. Initially, blood tubes were centrifuged at 2000 g for 10 min at room temperature to pellet cells while retaining plasma in the supernatant. Plasma was carefully transferred to new tubes without disturbing the buffy coat and subjected to a second high-speed centrifugation at 3000 g for 10 min to remove residual cellular debris. The resulting plasma was aliquoted and stored at − 80 °C until analysis.

## Spectrometry

Elemental compositions and serum copper isotope compositions were analyzed following the protocol described by Yang et al.^2^ The procedure involves the digestion of the sample by heating it overnight in nitric acid and hydrogen peroxide. After redissolution, a fraction is harvested for elemental analysis, performed on a Thermo Element 2 ICP-MS. The remainder of the fraction is double-spiked with ^57^Fe–^58^Fe, ^64^Zn–^67^Zn, and ^110^Cd–^111^Cd of known isotopic composition and is then used for metal separation by anion exchange chromatography using AG-MP1 resin. ^69^Ga–^71^Ga is added to the copper fraction at roughly twice the concentration of Cu for use in correcting instrumental mass bias (a universal issue in MC-ICP-MS). Standards are also measured routinely (every 4–6 samples) to correct for systemic drift during sessions. Isotope measurements were performed on a Thermo Neptune Plus MC-ICP-MS at the Earth Sciences Department of the University of Southern California, following instrumental settings and data acquisition methods published by Takano et al.^23^

Copper isotope fractionation is reported using standard delta notation in parts per 1000 (‰), calculated as follows:

$$\:{\delta\:}^{65}Cu=\left(\frac{{\left(\frac{{}^{65}Cu}{{}^{63}Cu}\right)}_{sample}-{\left(\frac{{}^{65}Cu}{{}^{63}Cu}\right)}_{NIST\:SRM\:976}}{{\left(\frac{{}^{65}Cu}{{}^{63}Cu}\right)}_{NIST\:SRM\:976}}-1\right)\:x\:1000\:‰$$

This value indicates the relative deviation of the ^65^Cu/^63^Cu ratio in the measured sample compared to its value in the standard reference material NIST SRM 976. Previous reports show that the reproducibility of δ^65^Cu at the 95% confidence level is 0.05‰ (meaning a minimum of a 0.1‰ change is required for resolution by MC-ICP-MS), with natural variations not exceeding ± 3‰^9^.

### Statistical analysis

Statistical analysis and plot generation of all data were performed in RStudio version 2024.04.2 + 764.

The normality of the data was analyzed using a Kolmogorov-Smirnov test. All datasets were found to be normally distributed, and Welch’s *t*-tests were therefore used to find significant differences between δ^65^Cu, trace metal concentrations, and Cu/Zn ratios of healthy and cancer patients. Continuous variables are reported as means.

### Study population

The study population included 123 deidentified male individuals, with the population characteristics presented in Table 1. Patients with localized, M1, or de novo metastatic prostate cancer (de novo MPC) are characterized as having an earlier-stage malignancy. Localized patients have cancer limited to the extent of the prostate. M1 and de novo MPC patients were newly diagnosed to have disease spread outside of the prostate region (within 6 months of initial prostate cancer diagnosis in this study) and are generally treated with anti-androgen therapy. Patients with metastatic castration-resistant prostate cancer (mCRPC) are characterized as having a later-stage malignancy no longer responsive to anti-androgen therapy. Patients with biochemical recurrences (BCHM Recurrence) or recurrent metastatic cancer achieved clinical remission and then had rising levels of serum prostate-specific antigen or clinical recurrence of metastatic cancer, respectively. For subsequent analysis, Localized/M1/de novo MPC samples were merged on the basis that these patients had an earlier-stage or newly metastatic malignancy and were untreated at the time of sample collection with an anticipated survival of 5 or more years^24^. mCRPC patients have a metastatic malignancy and a lifespan averaging less than 2 years^25^. Recurrent Metastatic/BCHM recurrence patients were merged on the basis that they have a biochemical recurrence and/or clinical recurrence of malignancy after previous disease remission (with prostate resection), such patients have an average survival of more than 10 years^26^.

**Table 1:** Characteristics of the Study Population and Average δ^65^ Cu by Subgroup.

Patient subtype	Count	Prostate resected	Average δ⁶⁵Cu (‰)	Standard deviation (±)
Healthy control	21	No	− 0.16	0.15
Localized	19	No	0.03	0.22
M1	8	No	− 0.03	0.21
de novo Metastatic prostate cancer (de novo MPC)	10	No	0.02	0.15
Metastatic castration-resistant prostate cancer (mCRPC)	47	Yes	− 0.22	0.26
Biochemical recurrence	3	Yes	− 0.06	0.32
Recurrent metastatic	14	Yes	− 0.15	0.22
Total	123			

## Results

**Fig. 1:**
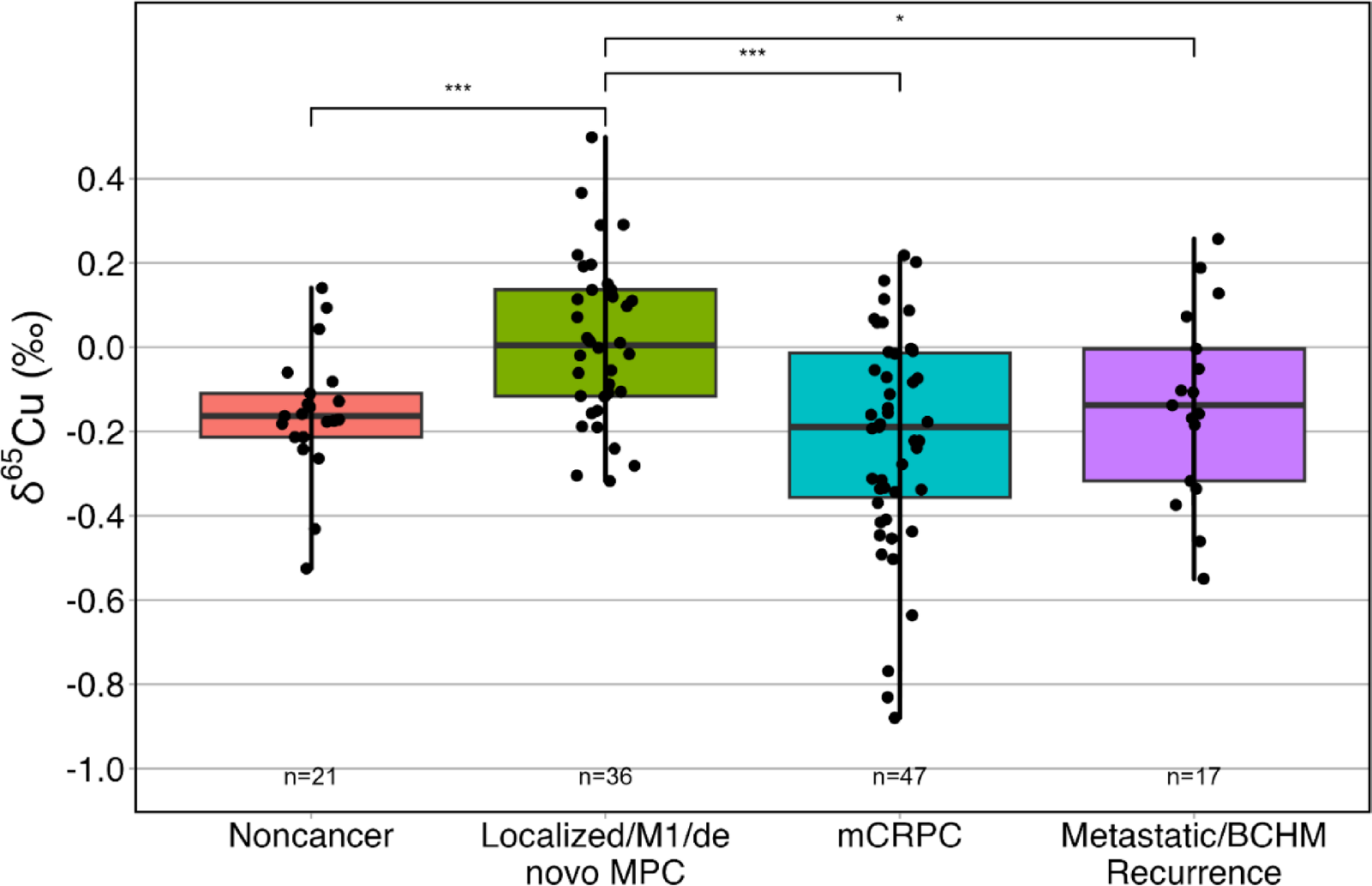
δ^65^Cu in male prostate cancer patients by prostate and cancer status. Patients with a lower burden of disease (localized/M1/de novo MPC) without prostate resections had serum significantly enriched in ^65^Cu compared to healthy controls, patients with a higher burden of disease (mCRPC), and patients with biochemical or clinical recurrences of disease. There were no other significant differences between groups. Boxes represent the 75% middle quantile, and whiskers the 95% quantile. *indicates *p* < 0.05, **indicates *p* < 0.01, and ***indicates *p* < 0.001 by Welch’s *t* test.

δ^65^Cu was measured for all patients, with results by subgroup shown in Table 1. After δ^65^Cu for each patient was measured, patients were regrouped based on the burden of their malignancy, and their values were compared. Figure 1 shows the result of the δ^65^Cu comparison between the new subgroups. Relative to healthy controls, prostate cancer patients in the earlier stages had a significantly higher average serum δ^65^Cu (− 0.16‰ vs. 0.01‰, *p* = 0.00047), indicating isotopically heavy samples. Prostate cancer patients with surgical resection of the prostate (all in the late stages of the disease) showed an average δ^65^Cu significantly lower than early-stage patients (0.01‰ vs. − 0.22‰, *p* = 0.00001) but not significantly different from healthy controls. Patients with biochemical or clinical disease recurrences do not differ significantly from the healthy controls.

**Fig. 2:**
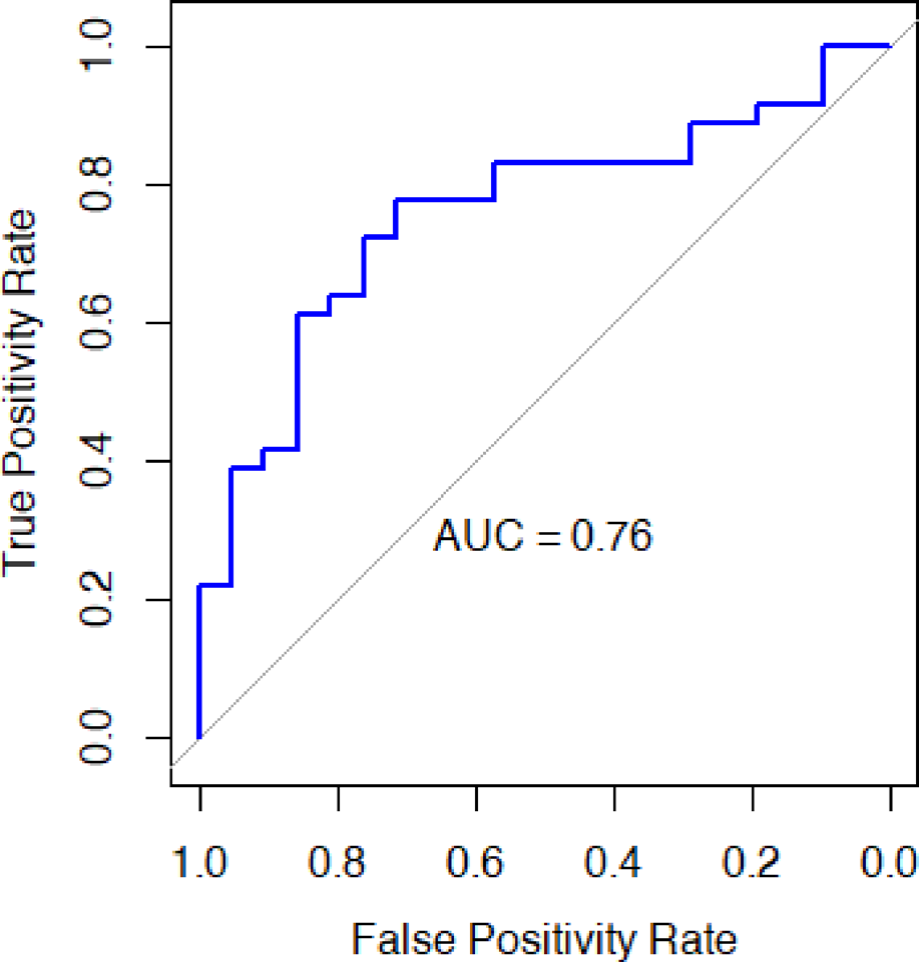
Receiver Operator Characteristics curve of δ^65^Cu in the plasma of localized/M1/de novo MPC patients predicting against healthy controls. The area under the curve (AUC = 0.76) reflects moderate discrimination between earlier-stage cancer and control groups based on isotopic composition.

A Receiver Operating Characteristics (ROC) curve was then generated to analyze the δ^65^Cu data (Fig. 2). ROC curves are used to assess the diagnostic performance of medical tests and predictive models, evaluating the probability that a result obtained is a true or false positive. A higher value for the area under the curve (AUC) indicates a higher true positivity rate for a given false positive rate, normally ranging from 0.5 (performance no better than random chance) to 1 (perfect discrimination between groups). The AUC for δ^65^Cu in the plasma of localized/M1/de novo MPC patients against healthy controls was 0.76, indicating moderate discrimination. However, it is ineffective in detecting high-burden disease or biochemical/clinical recurrence.

**Fig. 3:**
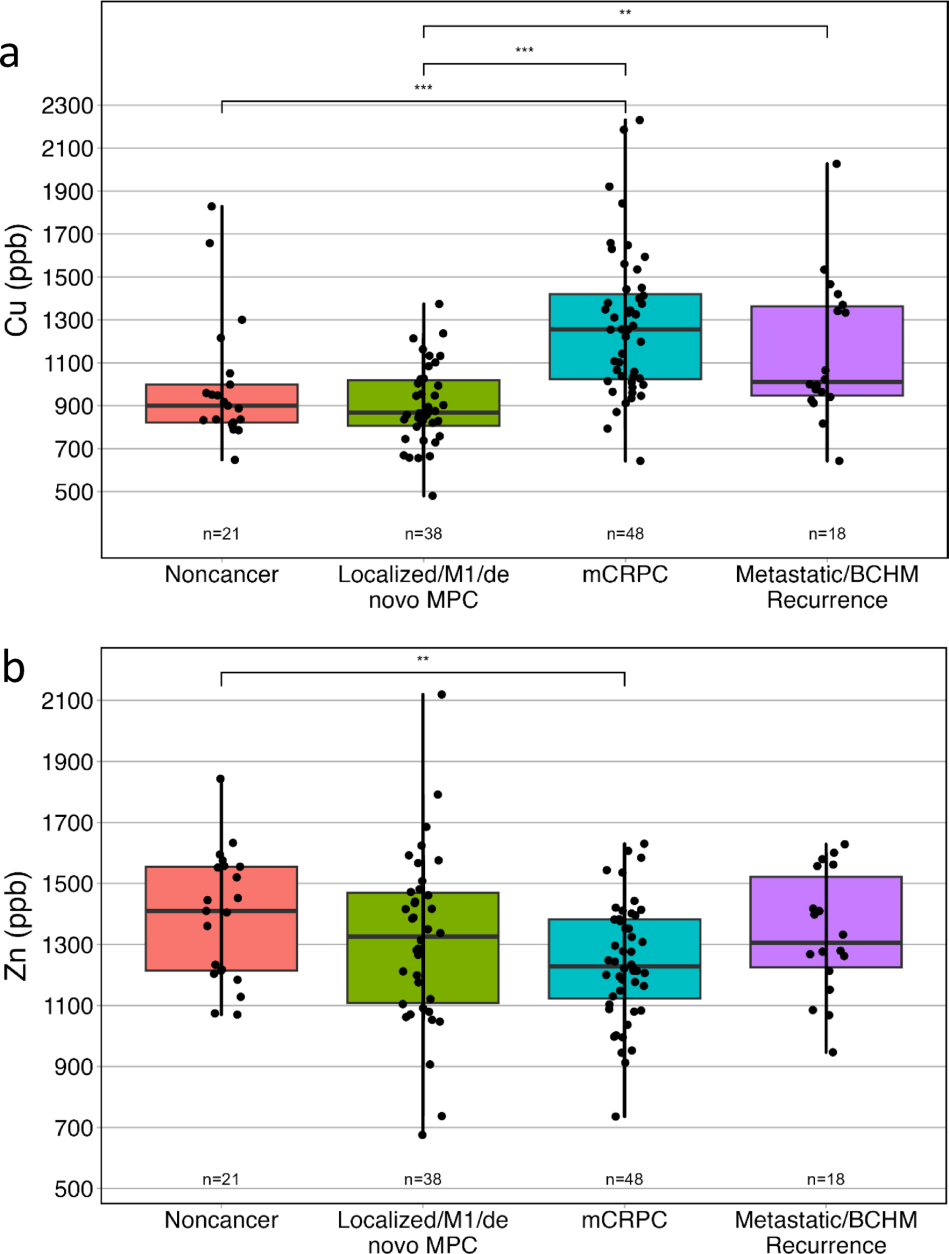
Copper and zinc concentrations in male prostate cancer patients by prostate and cancer status. (**a**) Patients with mCRPC have significantly higher serum copper than healthy controls or earlier-stage patients. (**b**) Conversely, zinc concentrations trend downward, with mCRPC patients having significantly lower serum zinc concentrations than healthy controls. Boxes represent the 75% middle quantile, and whiskers the 95% quantile. *indicates *p* < 0.05, **indicates *p* < 0.01, and ***indicates *p* < 0.001 by Welch’s *t* test.

Copper and zinc concentrations were similarly measured, with results shown in Table 2. In this study population, mean serum copper concentrations fell within the international reference range (804–1620 ppb) for all groups. However, average serum zinc concentrations in all groups exceeded the international reference range (785–1046 ppb). Copper and Zn concentrations were classified according to international reference values (Table 2). Although most patients within each group fell within the international reference range for Cu concentration, significant differences in concentration were observed between groups (Fig. 3). Patients with mCRPC displayed significantly higher serum Cu than healthy controls and patients with earlier-stage disease (1270 ppb vs. 990 ppb, *p* = 0.0002, and 904 ppb, *p* = 0.0001, respectively). Conversely, serum Zn concentrations were significantly lower in patients with mCRPC as compared to healthy controls (1243 vs. 1392 ppb, *p* = 0.01). This reciprocal interaction has been previously described in the literature^27^.

**Table 2:** Average serum copper and zinc concentrations by subgroup and distribution relative to international reference ranges.

Subtype	Mean Cu ± SD (ppb)	Cu ≤ 804 (%)	804 < Cu < 1620 (%)	Cu ≥ 1620 (%)	Mean Zn ± SD (ppb)	Zn ≤ 785 (%)	785 < Zn < 1046 (%)	Zn ≥ 1046 (%)
Healthy control	990 ± 291	19%	71%	10%	1392 ± 210	0%	0%	100%
Localized/M1/de novo MPC	904 ± 187	26%	74%	0%	1316 ± 280	5%	3%	92%
mCRPC	1277 ± 335	4%	81%	15%	1243 ± 193	2%	15%	83%
Recurrent metastatic/BCHM recurrence	1153 ± 331	6%	89%	6%	1335 ± 200	0%	6%	94%

**Fig. 4:**
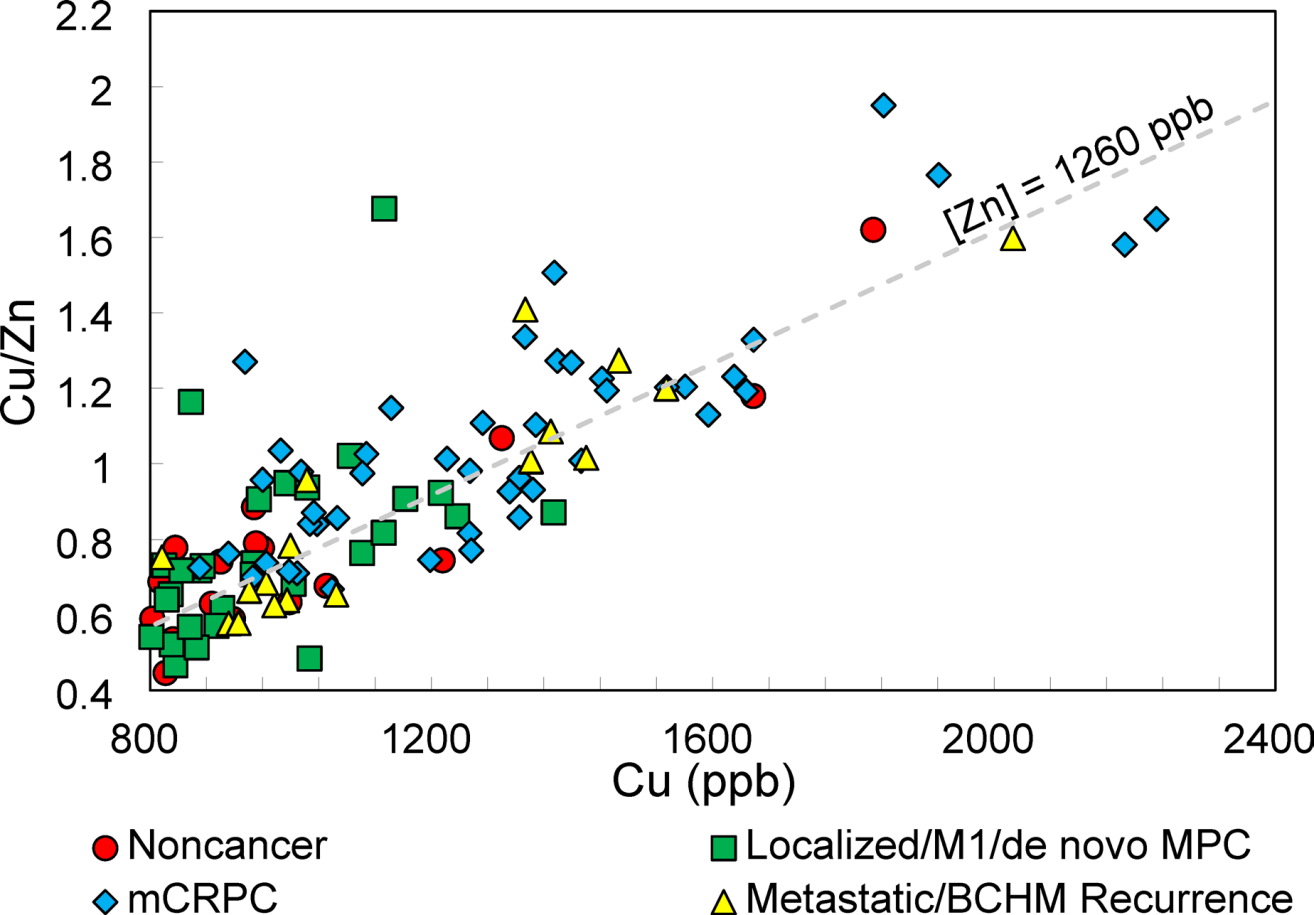
Correlational analysis of Cu to Zn ratios versus Cu concentrations in patient subgroups. R^2^ for noncancer is 0.84, Localized/M1/de novo MPC is 0.36, mCPRC is 0.67, and metastatic/BCHM recurrence is 0.82. Healthy controls and patients with biochemical/clinical recurrence showed less variability and, therefore, greater regulation of the Cu/Zn ratio, as demonstrated by tighter adherence to their respective trendlines.

**Fig. 5:**
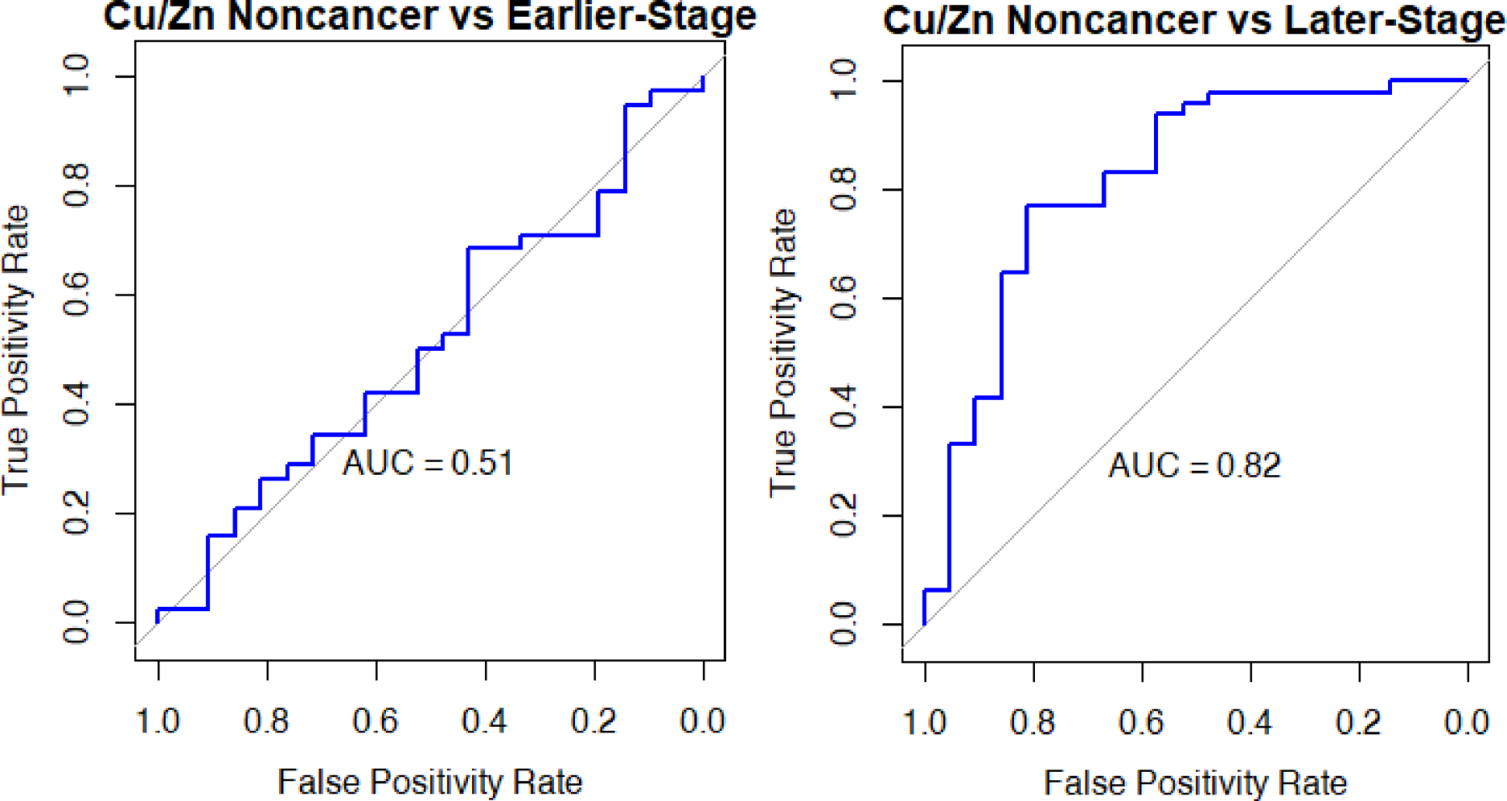
Receiver Operator Characteristics (ROC) curve of Cu/Zn ratios in the plasma of localized/M1/de novo MPC (left) and mCRPC patients (right) against healthy controls. The areas under the curve (AUC = 0.51 and 0.82, respectively) indicate greater separation between groups at higher disease burden and minimal distinction at lower burden.

Correlational analysis of Cu/Zn ratios versus Cu concentrations was then performed and compared between patient subgroups (Fig. 4). The healthy controls show a clear correlation representing the strict regulation of Zn levels in the serum (R^2^ = 0.84), while earlier-stage and mCRPC patients demonstrated greater variability consistent with deregulation (R^2^ = 0.36 and 0.67, respectively). Those with biochemical/clinical recurrences of disease maintained strict regulation of Cu/Zn (R^2^ = 0.82). The Cu/Zn ratio was then analyzed with an ROC curve to determine the predictive power of this measurement (Fig. 5). The Cu/Zn ratio was shown to be ineffective in differentiating between noncancer and lower-burden-of-disease patients (AUC = 0.51) but was reasonably accurate in discriminating between healthy controls and higher-burden disease (AUC = 0.82).

## Discussion

This study demonstrates the successful application of high-precision copper isotope and Cu/Zn ratio analysis, techniques rooted in geochemistry, to human plasma for the study of prostate cancer-associated metabolic changes across stages for the first time. Translating these methods to human biology establishes a proof-of-principle for their use in clinical samples, showing that stable isotope fractionation can resolve subtle biochemical differences among patients using milliliter amounts of human serum and identifying an important signal in certain populations.

### Copper isotope fractionation

The δ⁶⁵Cu results differed from previous reports in other malignancies, which have typically described lighter serum Cu isotopic compositions in the presence of disease. The main finding regarding δ^65^Cu in the present study was that patients with a lower burden of disease who have not undergone treatment (cancer localized to the prostate gland or newly diagnosed early metastatic disease) have a δ^65^Cu level higher than that of healthy controls and those with mCRPC (Fig. 1). The AUC of 0.76 reflects moderate discrimination between patients with earlier-stage disease and healthy controls (Fig. 2). Patients with late-stage disease had wide variability of δ^65^Cu values that did not differ significantly from controls (Fig. 1). These results stand in contrast to previous observations of various disease states but align with the unique metabolic profile of many malignancies of the prostate. As discussed, several mechanisms have been proposed in the literature for the fractionation pattern seen in other diseases. These include increased tumor Cu uptake under hypoxic conditions, hepatic fractionation during altered metal metabolism, and changes in metabolism related to multisystem disease^5,7,8,12,14^. These previous proposals, however, cannot fully explain the patterns seen in this study and were developed in the context of the later stages of disease.

One key confounder in our dataset that is unique to prostate cancer is the role of anti-androgen therapy. Depletion of testosterone production in the gonads is a mandatory component of care in patients with mCRPC, as well as for many of the relapsed patients with earlier-stage disease. However, those patients with de novo untreated prostate cancer would have had normal testosterone levels at the time of study enrollment. Testosterone, primarily through androgen receptor (AR)-mediated mechanisms, influences the expression and activity of key proteins involved in copper (Cu) and zinc (Zn) homeostasis^28,29^. Metallothioneins 1–3 are low molecular weight proteins that bind both Zn and Cu ions and are lost under castrate conditions. Zinc transporters such as ZiIP0 (SLC39A9) bind testosterone with high affinity, activate G proteins, and elevate intracellular free Zn via influx/efflux from organelles^30^. Superoxide dismutase also may play a role in the oxidative status of the tumor and is diminished in the setting of androgen depletion. This has the potential to impact isotope use in patients who are under conditions of androgen deprivation despite having a relatively high tumor burden^31^. Thus, when considering the role of a plasma assay for divalent metal cations, the impact of sex steroids and other physiologic conditions impacting cation use must be accounted for.

Overall, the observed isotopic patterns likely arise from the interaction of multiple biological processes rather than a single mechanism, with the dominant mechanism(s) being unclear. Tumor‑intrinsic factors such as altered metabolic fluxes, hypoxia‑driven metal uptake, and redox‑dependent binding environments may influence copper utilization within cancer cells. At the same time, systemic processes, including hepatic copper handling, inflammatory responses, and endocrine regulation by sex steroids as discussed above, can modify the circulating copper pool from which isotopic measurements are derived. Consequently, the serum δ^65^Cu signal should be interpreted as an integrated reflection of both tumor biology and systemic physiological state.

### Cu/Zn concentrations and ratios

As discussed, it is well documented that serum copper levels are elevated, and conversely, zinc levels are decreased in prostate cancers^17^. Increased copper uptake by prostate cells has even been utilized as a means of imaging prostate tumors, which has been explored as a drug target^32,33^. As previously described, copper plays roles in maintaining iron oxidation states, controlling electron fluxes, and removing reactive oxygen species. The proliferation of prostate cells relies on copper and its homeostasis. The reciprocal relationship between copper and zinc uptake has also been well documented^27^.

The present study corroborates these findings and, in addition, shows that a significant increase in serum copper (and, conversely, a significant decrease in serum zinc) does not occur until high-burden disease (Fig. 3). This dysregulation may be visualized by changes in the serum copper-to-zinc ratio (Fig. 4). ROC curve analysis displays this clearly; the Cu/Zn ratio proves ineffective (AUC = 0.51) in predicting earlier-stage disease but shows good discrimination in identifying later-stage disease (AUC = 0.82) (Fig. 5). This pattern suggests that copper and zinc concentrations remain relatively stable until high-burden disease, after which broader metabolic disturbances become evident. The Cu/Zn ratio, therefore, appears to capture cumulative physiological changes rather than stage-specific effects.

Zinc concentrations in all cohorts were modestly elevated relative to commonly cited clinical reference ranges. Because detailed dietary or supplementation histories were not available, it remains unclear whether this reflects increased intake, use of zinc-containing supplements, altered metabolic handling, or systemic physiological changes. This observation is notable given the interconnectedness of zinc and copper uptake and their role in nutrition-redox-inflammation axes, as a variety of redox-active enzymes depend on copper and/or zinc^6^. Recent studies have reported that higher serum zinc levels may be associated with improved survival across several cancer types, whereas elevated copper concentrations may correlate with poorer outcomes. Variation in their circulating concentrations has been proposed as an integrative indicator of physiological stress and nutritional status rather than a cancer-specific biomarker alone^34^. Because similarly elevated zinc concentrations were observed in the control cohort, the absolute values reported here cannot be attributed to the disease state alone; however, the observed downward trend across disease stages is consistent with previously reported changes in zinc metabolism in prostate cancer, emphasizing the importance of considering relative changes alongside baseline trace-metal variability.

### Limitations

Limitations of this study include a modest sample size, single plasma measurements per patient, and a lack of matched tissue or functional data to confirm the physiological origin of the observed metal shifts. Furthermore, heterogeneity in treatment history, comorbidities such as cirrhosis, and nutritional status may have influenced copper and zinc levels, particularly in late-stage patients. Further studies with larger, clinically stratified cohorts and paired tissue-plasma analyses are needed to both confirm these observations and to define how isotope-based approaches can enhance our understanding of human metabolism and disease.

## Conclusion

This study demonstrates that high-precision copper isotope and Cu/Zn ratio measurement techniques adapted from geochemistry can detect measurable differences in trace-metal composition among patients with prostate cancer. The δ^65^Cu patterns observed here, namely heavier serum copper in earlier-stage disease and wide variability in late-stage disease, differ from those reported in other malignancies, which often show lighter serum Cu isotopic compositions in high burden disease, perhaps due to unique factors associated with androgen deprivation therapy in prostate cancer patients. The unique metabolic characteristics of prostate cancer appear to produce distinct fractionation behavior. Cu/Zn ratios remained stable in early disease but increased in advanced stages, capturing the cumulative effect of changes that occurred due to the cancer burden overall. These findings highlight both the sensitivity of isotope-based measurements in highly dilute plasma and the biological diversity of metal metabolism across tumor types. Because of the limitations of the study, including a relatively small number of samples and potential heterogeneity between patient comorbidities and nutritional status, the observed isotopic and elemental trends should be interpreted as probabilistic indicators of altered metal metabolism rather than definitive diagnostic signatures. Larger, prospectively designed studies will be necessary to validate the reproducibility and clinical significance of these findings. Although the precise mechanisms underlying these isotopic shifts remain uncertain, the results confirm that prostate malignancy and its associated physiological changes, including organ dysfunction, treatment impact, and altered redox balance, affect copper and zinc homeostasis.

## Data Availability

All deidentified data reported in this paper will be shared by the lead contact upon request. This paper does not report original code. Requests for further information and resources should be directed to and will be fulfilled by the lead contact, Robert Raad (raadr@usc.edu).
